# Electrochemical immunosensor based on carboxylated single‐walled carbon nanotube‐chitosan functional layer for the detection of cephalexin

**DOI:** 10.1002/fsn3.1382

**Published:** 2020-01-09

**Authors:** Wenlong Yu, Yaxin Sang, Tianying Wang, Weihua Liu, Xianghong Wang

**Affiliations:** ^1^ Faculty of Food Science and Technology Agricultural University of Hebei Baoding China

**Keywords:** cephalexin, competitive immunoreaction, electrochemical immunosensor, nanomaterials, SWNTs

## Abstract

In this study, a sensitive and selective electrochemical immunosensor for cephalexin (CEX) determination on a glassy carbon electrode (GCE) surface was modified by a carboxylated single‐walled carbon nanotubes/chitosan (SWNTs‐COOH/CS) composite. The SWNTs‐COOH/CS composite was used to enhance sensor performance and to enlarge the electrochemical response of CEX. The cephalosporin‐ovalbumin coupling (CEX‐OVA) was synthesized using the reactive ester method. The free CEX in solution could be effectively measured based on the competitive immunoreaction between CEX‐antibody and CEX. Under optimal conditions, the electrochemical immunosensor offered an excellent response for CEX. The linear range was 1–800 ng/ml, with a detection limit of 45.7 ng/ml (*S/N* = 3). This method was applied to determine CEX in six different samples and obtained the recovery range from 80.15% to 94.04%. These results indicated that the fabricated electrochemical immunosensor and sensing method are suitable for quantification of CEX in real samples. These have great potential for wider applications in environmental and agri‐food products industries.

## Graphical abstract

This study described a sensitive, specific, and simple electrochemical immunosensor for detection of CEX by integrating SWNTs‐COOH/CS with enzymatic signal readout. The sensor might further furnish a versatile and forceful tool to guarantee food safety.

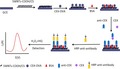

## 1 INTRODUCTION

Cephalexin (CEX) belongs to the first generation of synthetic semisynthetic cephalosporin antibiotics. Its mechanism of action works through the inhibition of the synthesis of the bacterial cell wall during bacterial division and letting the bacteria die under the influence of external osmotic pressure. Compared with several other types of cephalosporin, cephalexin has the advantages of being inexpensive and is orally absorbed, etc. It has been widely employed in veterinary clinics and for the treatment of urinary tract infections, skin infections, and mastitis in dairy cows (Sawant, Sordillo, & Jayarao, 2005). In the European Union (EU), maximum residue limits (MRL) for CEX in milk, muscle, fat, liver, and kidney are 100, 200, 200, 200, and 1,000 μg/kg, respectively (European Agency for the Evaluation of Medical Products, 1999). However, with continuously increasing the complications of animal epidemics, as well as the non‐compliance with the stipulations during withdrawal periods, the cephalexin residues in animal foods were consequently becoming more severe. In particular, excessive residues in milk have been endangering human health and food safety issues, and affecting the export trade severely (Xie et al., 2009; Zhi, Meyer, Bedem, & Meusel, 2001). Studies have shown the phenomenon of over‐standard use of cephalosporin antibiotics in livestock and poultry farms. Therefore, sensing the presence of CEX in food samples is of great importance not only for consumer exposure assessment on CEX but also for identifying the abuse of the antibiotic CEX.

Traditional detection methods include microbiological methods, spectrophotometry, high‐performance liquid chromatography (HPLC), and high‐performance liquid chromatography‐mass spectrometry (HPLC‐MS). However, these methods inevitably count on advanced apparatuses being available to deal with complicated operations, and it takes a long time to complete (Cazorla‐Reyes, Romero‐González, Frenich, Rodríguez Maresca, & Martínez Vidal, 2014; Han et al., 2012; Zhang et al., 2013). Therefore, it is necessary to develop simple, rapid, sensitive, and low‐cost methods for sensing CEX residue in food products.

Electrochemical immunosensor combines specific ELISA with highly sensitive sensing technology, good selectivity, and fast analyzing ability (Gayathri, Mayuri, Sankaran, & Kumar, 2016; Jing et al., 2016; Zhang, Li, Ma, Zhang, & Zheng, 2016). In recent years, the rapid development of nanomaterials technologies have drawn much research attention. Single‐walled carbon nanotubes, one of the nano‐materials, were made of a grapheme monolayer which has good physical and chemical properties. Furthermore, it is considered to be a high‐quality sensor signal (Cao, Zhang, Chai, & Yuan, 2013; Tong et al., 2016; Yu et al., 2015). Electrochemical immunosensor, single‐walled carbon nanotubes (SWNTs) were introduced to enhance the ability of electron transfer, biocompatibility of the working electrode surface, and the sensitivity of the electrochemical immunosensor method (Zhang, Li, Wang, et al., 2016). Yang et al. (2016) using ultrasound to uniformly disperse single‐walled carbon nanotubes in chitosan to form a composite membrane, modified the surface of the electrode to construct an electrochemical immunosensor. This was done to detect fumonisin B_1_, whereby the linearity is good, the minimum detection limit is 2 g/ml, which is much lower than the EU limit of 2–4 mg/L. Furthermore, recovery and reproducibility is better and can be applied to the actual sample subjected to fumonisin B_1_ detection (Yang et al., 2016). The work done by Zhang et al. presents the carbon nanotubes modified on the electrode surface‐immobilized antigen coated with nano‐gold labeled secondary antibody. The hydrochloric acid that occurs in electrical oxidation, resulted in changes in the signal to complete the microsystem. The linear range was 0.0025–5 μg/L, and the lowest detection limit was 1.68 ng/ml. Both indicated good accuracy and reproducibility, and there a wide range of applications in environmental monitoring emerged (Zhang, Kang, Hao, Lu, & Cheng, 2015). The present study successfully produced an electrochemical immunosensor for the rapid detection of cephalexin in animal foods. This method was more sensitive than other methods and has good reproducibility and satisfactory results.

## 2 MATERIALS AND METHODS

### 2.1 Materials

N‐hydroxysuccinimide (NHS), N‐(3‐dimethylaminopropyl)‐N′‐ethylcarbodiimide hydrochloride (EDC), Cephalexin (CEX), Cefradine (RAD), Cefadroxil (CER), Cefoperazone (PER), Cefazolin (ZOL), Cefotaxime (TAX), and Cefuroxime (CFX) were purchased from Shanghai Yuan Ye Biological Technology Co., Ltd. HRP‐anti‐antibody, ovalbumins (OVA), and chitosan (CS) were provided by Sigma‐Aldrich. SWNTs‐COOH (<5 nm diameter) was purchased from Shenzhen Nanotech Port Ltd. Co. Hydroquinone (HQ) and hydrogen peroxide (H_2_O_2_) were purchased from Shanghai Yuan Ye Biological Technology Co., Ltd. Potassium chloride (KCL) and potassium ferricyanide (Fe_3_(CN)_6_) were purchased from the National Group Chemical Reagent Co., Ltd. Voltammetric experiments were performed in freshly prepared phosphate buffer solution (PBS, 0.01 mol/L). All solutions were prepared with deionized water.

### 2.2 Apparatus

The differential pulse voltammetry (DPV), and cyclic voltammetry (CV) measurements were carried out on CHI 660e electrochemistry workstation (Shanghai CH Instruments), connected to a computer. The morphology and particle sizes of the samples were estimated from scanning electron microscope (SEM) (Hitachi 600). All experiments were performed with a conventional three‐electrode system. The modified glassy carbon electrode (GCE) was used as the working electrode, a platinum wire as the counter electrode, and Ag/AgCl (sat. KCl) as the reference electrode.

### 2.3 Preparation of immunosensor

The electrode was first polished with 0.3 μm alumina, ultrasonically cleaned in sulfuric acid, absolute ethanol, deionized water, and dried at room temperature. CEX‐OVA was prepared using the active ester method. SWNTs‐COOH/CS (1 mg/ml) nano‐composites were prepared using ultrasonication of SWNTs‐COOH, chitosan, and 1% acetic acid solution (Zhang, Wang, Qiuwei, & Shuguo, 2016).

The prepared SWNTs‐COOH/CS/GCE electrode was washed with phosphate buffer solution (1× PBS, 0.01 M, pH 7.4). After that, the electrode was immersed in the freshly made activation solution of EDC/NHS, at concentrations of 5 mM and 2 mM, respectively. It was then incubated at 37°C for 50 min to activate the carboxyl groups on SWNTs‐COOH. Following this, the activated electrode was again thoroughly washed with 1× PBS buffer. A total of 10 μl CEX‐OVA (5 μg/ml) was immediately dropped vertically onto the electrode surface by a pipette gun and incubated at 37°C for 50 min. After incubation was complete, the prepared electrode was washed with PBS buffer.

About 10 μl of 1% bovine serum albumin (BSA) was added to the surface of CEX‐OVA/SWNTs‐COOH/CS/GCE and kept for 1 hr to block the unreacted active sites on the electrode surface. Finally, the prepared electrodes were thoroughly washed with PBS buffer to obtain CEX‐OVA/SWNTs‐COOH/CS/GCE, which was then stored in a refrigerator at 4°C for later use.

About 5 μl of PBS (pH 7.4)‐diluted CEX in the range of 0–1,000 μg/ml was evenly mixed with 5 ml of anti‐CEX (diluted at 1:160). The mixture was added to the surface of CEX‐OVA/SWNTs‐COOH/CS/GCE electrode and incubated at 37°C for 50 min to obtain anti‐CEX/CEX‐OVA/SWNTs‐COOH/CS/GCE. During the reaction, CEX‐OVA fixed on the electrode competed with free CEX in the mixed solution to produce a certain amount of anti‐CEX.

**Scheme 1:**
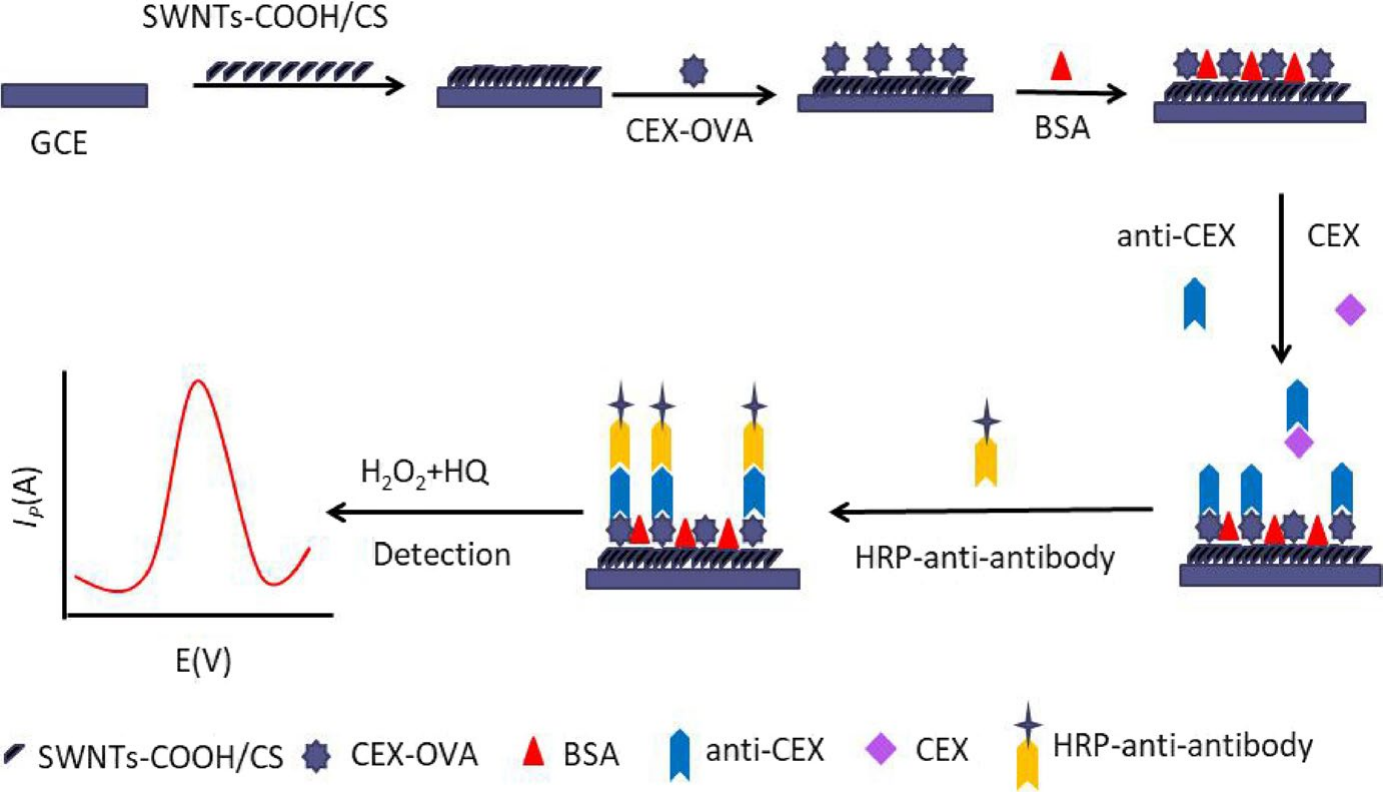
The schematic diagram of the fabrication of the immunosensor

The electrode was washed with phosphate buffer solution (1× PBS, pH 7.4). About 10 μl of 160‐fold diluted horseradish peroxidase was dropped vertically using a pipette gun to label goat anti‐rabbit IgG(H + L) secondary antibody and then incubated at 37°C for 50 min. Then, the prepared buffer solution of 3 mM H_2_O_2_ and 3 mM HQ (1:1, v/v) were used as the immune response buffer system, in which the working electrode was placed. The saturated calomel electrode was used as the reference electrode, and the platinum wire electrode was used as the counter electrode, which was determined using differential pulse voltammetry (DPV). Scheme 1 shows the detailed production of the sensor.

### 2.4 Sample preparation

Milk‐derived and meat products are purchased at local supermarkets. The dairy products were put accurately into the polystyrene centrifuge tube, and 35 ml acetonitrile was added. It was then mixed and subjected to ultrasonic extraction for 30 min. Then, it was cooled to room temperature, followed by adding 200 g/L zinc acetate 2.0 ml, acetonitrile constant volume to 50 ml, vortexed for 0.5 min, subjected to 15,000 g centrifugation for 5 min, collection of supernatant (10 ml), 15,000 g and then centrifuged for 5 min. The supernatant was collected into a 0.22 μm filter and diluted 10 times by PBS solution before use.

It is important to obtain a proper‐sized meat sample and stir it well. Then, it is extracted for 15 min at room temperature with 1:1 (v:v) methanol/water (8:2), centrifuged at 2,400 g for 15 min and a supernatant is created through an 0.45 μm organic filter and diluted 20 times with a PBS solution before use.

## 3 RESULTS AND DISCUSSION

### 3.1 SEM characterization of SWNTs‐COOH, CS and SWNTs‐COOH/CS nano‐composites

**Figure 1:**
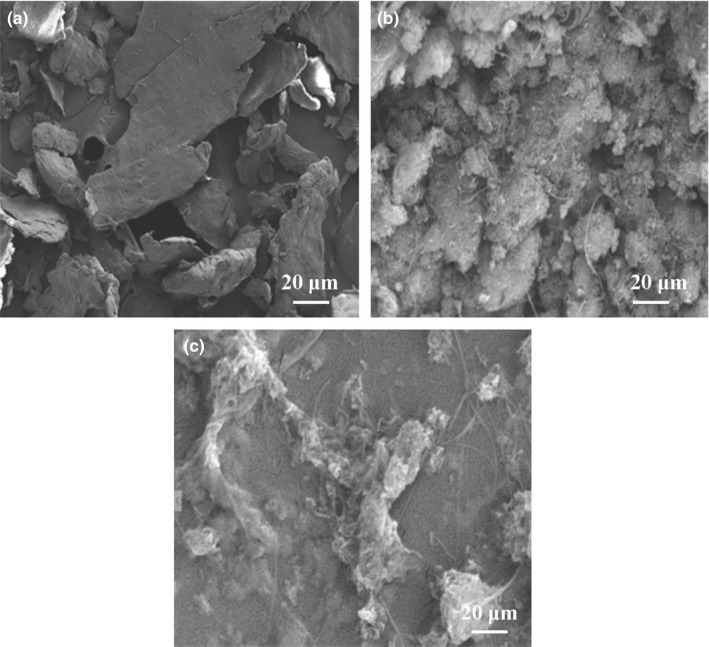
The SEM image of (a) SWNTs‐COOH, (b) CS, and (c) SWNTs‐COOH/CS

The morphologies and microstructures of the as‐prepared different modified films were investigated by using SEM (Figure 1). The results in Figure 1a show that the structure of SWNTs‐COOH film is a smooth sheet. Figure 1b illustrates the loose cluster structure of CS. Figure 1c depicts a good dispersion structure when the CS was embedded steadily and evenly on the surface of SWNTs‐COOH. This phenomenon suggests the successful adsorption of the CS on the SWNTs‐COOH surface through carboxyl affinity. Furthermore, the SWNTs‐COOH/CS can significantly increase the sensitivity of the sensor by providing greater biocompatibility and a specific surface area.

### 3.2 XRD characterization of SWNTs‐COOH/CS structure

**Figure 2:**
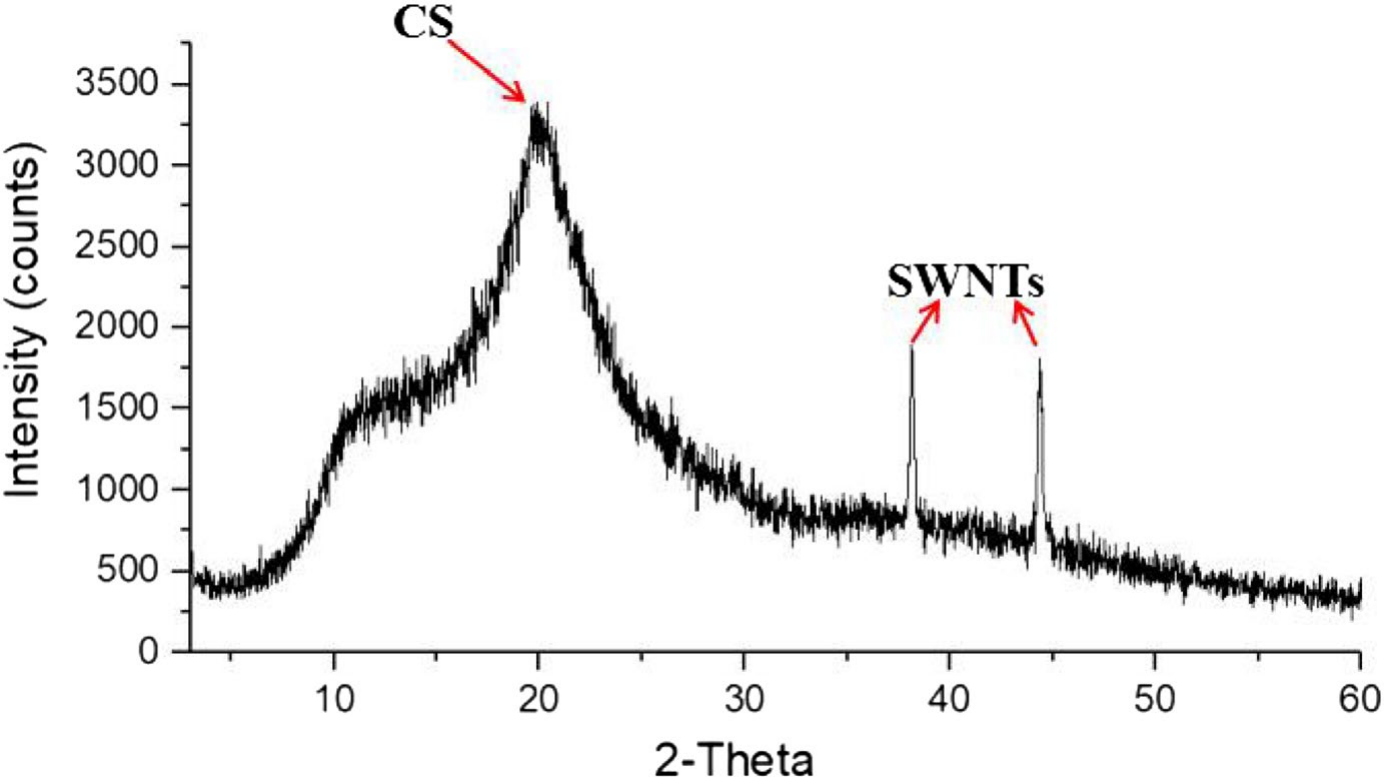
XRD characterization of SWNTs‐COOH/CS structure

The structure of SWNTs‐COOH/CS was characterized by XRD. The results are shown in Figure 2. In the spectra of the complexes, the 2θ characteristic peaks of CS appeared between 10° and 20°, and the two 2θ characteristic peaks of SWNTs appeared between 35° and 45°. This indicated that CS and SWNTs‐COOH were well combined and the synthesis of SWNTs‐COOH/CS was successful.

### 3.3 Electrochemical characterization of the immunosensor

**Figure 3:**
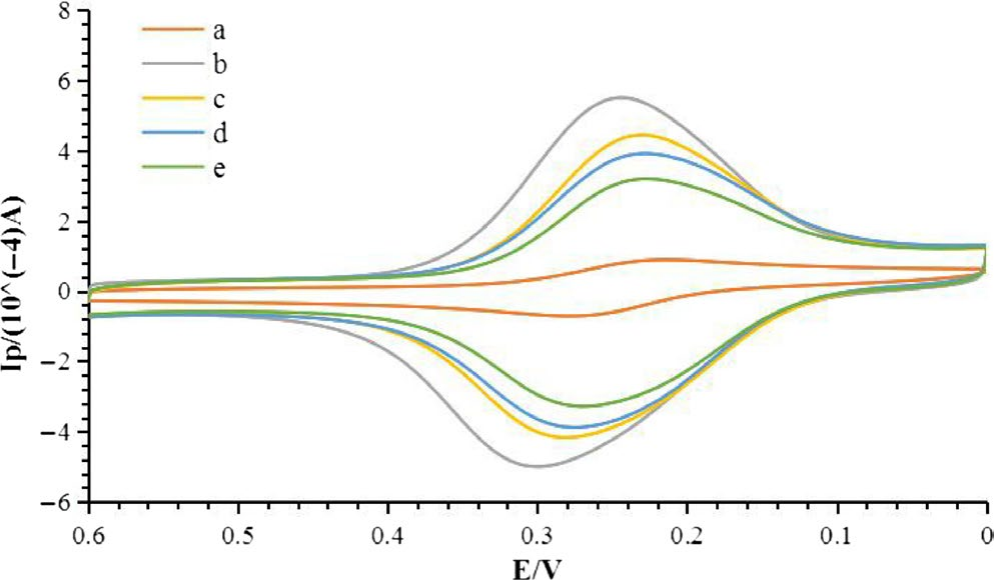
The CV in solution consisting 1 mmol/L Fe_3_(CN)_6_ and 0.1 mol/L KCl. (a) GCE; (b) SWNTs‐COOH/CS/GCE; (c) CEX‐OVA/SWNTs‐COOH/CS/GCE; (d) anti‐CEX/CEX‐OVA/SWNTs‐COOH/CS/GCE; and (e) HRP‐anti‐antibody/anti‐CEX/CEX‐OVA/SWNTs‐COOH/CS/GCE

The electrochemical performances of GCE, SWNTs‐COOH/CS/GCE, CEX‐OVA/SWNTs‐COOH/CS/GCE, anti‐CEX/CEX‐OVA/SWNTs‐COOH/CS/GCE and HRP‐anti‐antibody/anti‐CEX/CEX‐OVA/SWNTs‐COOH/CS/GCE were tested using CV in PBS solution (pH 7.4), which served as the electrochemical probe. As shown in Figure 3, the peak current of GCE is low (curve a). After being coated with SWNTs‐COOH/CS, a significant increase in the redox peak currents can be observed (curve b), owing to the excellent electron transfer ability of SWNTs and CS. This result proves that SWNTs‐COOH/CS can enlarge the electrochemical reaction to a large extent. In contrast, a noticeable reduction in the peak current is clearly observed on the CEX‐OVA/SWNTs‐COOH/CS/GCE (curve c). This phenomenon indicates that the CEX‐OVA is an obstacle and therefore significantly blocked the transport of electrons and the spread of the redox probe to the electrode interface. After the specific binding with antibody and HRP‐anti‐antibody, the peak current decreased due to specific immune responses, antibody, and HRP‐anti‐antibody blocked electron transport as proteins (curve d and curve e). This indicates that the HRP‐anti‐antibody has been successfully immobilized on the electrode surface.

### 3.4 Optimization of experimental conditions

**Figure 4:**
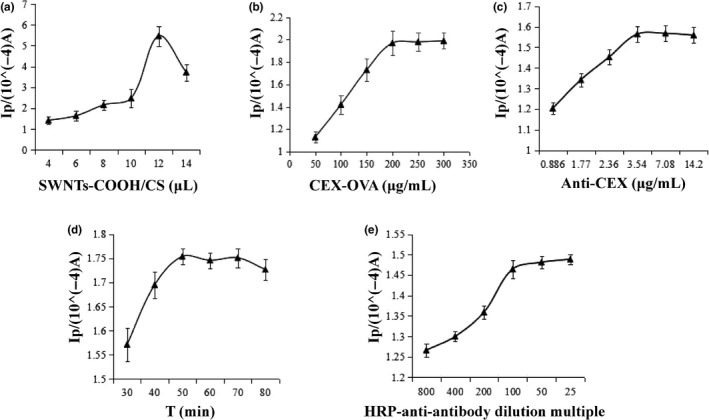
Dependences of DPV peak currents on (a) amount of SWNTs‐COOH/CS, (b) dilution ratio of anti‐CEX, and (c) incubation time, when one parameter changes while the others are unaltered. (d) HRP‐anti‐antibody dilution multiple

In order to achieve excellent analytical capabilities, some of the key parameters of the test were optimized. As shown in Figure 4a, the peak current of the CV increased gradually, when SWNTs‐COOH/CS was between 4 and 12 μl. This could possibly be caused by the excellent electron transfer ability of SWNTs‐COOH/CS. The peak current reached the maximum value of (5.46 × 10^–4^ A) when SWNTs‐COOH/CS was 12 μl. An excessive amount of SWNTs‐COOH/CS gathering on the electrode surface will impede the transfer of electrons. Therefore, 12 μl was used as the optimal amount of SWNTs in this work. As shown in Figure 4b, the peak current detected by DPV increases when the CEX‐OVA concentration also increases. When the concentration reaches 200 μg/ml, the peak current tends to be stable, so the optimal working concentration of CEX‐OVA is 200 μg/ml. The concentration of anti‐CEX in the incubation solution is also an important parameter in competitive format due to a fixed amount of CEX‐OVA domain at the electrode interface and a competitive immune response to the binding of free CEX to anti‐CEX in solution. In order to obtain the optimal concentration of anti‐CEX, the prepared CEX‐OVA/SWNTs‐COOH/CS/GCE was incubated with different concentrations of anti‐CEX solution. As shown in Figure 4c, the peak current increased sharply as the concentration of anti‐CEX increased from 0.886 to 3.54 μg/ml. With a further increase in the concentration of anti‐CEX, the peak current then decreased slowly. This indicates that the saturation of binding sites is reached at 3.54 μg/ml. Thus, the concentration of the anti‐CEX is identified as 3.54 μg/ml in this operation. As we expected, the incubation time influences the peak current greatly and the experimental results are shown in Figure 4d. The peak current increased as time passed within the 30–50 min range. The competitive reaction between CEX‐OVA and anti‐CEX and CEX takes time to bind and form a stable immune product. The specificity binding was saturated when the reaction time lasted 50 min. Therefore, 50 min was used as the incubation time in this work.

The effect of HRP‐anti‐antibody dilution factor on the peak current of DPV is shown in Figure 4e, when HRP‐anti‐antibody is diluted 100 times, the peak current of DPV reaches the maximum and then gradually stabilizes. Therefore, 100 times is selected as the optimal dilution multiple.

**Figure 5:**
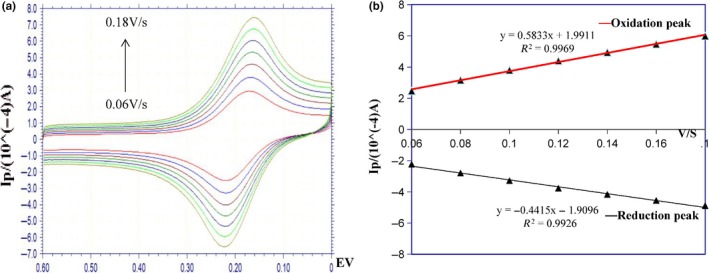
CV peak current at different scan rates. (a) CV response graph, (b) the currents of oxidation peak and reduction peak

The factors that influence the different scanning rates on the peak current of CV are shown in Figure 5. In the 5 mol/L K_3_ [Fe(CN)_6_] solution of 0.5 mol/L KCL, the scanning rates were 60, 80, 100, 120, 140, 160, and 180 mV/s in the voltage range of 0.55 ~ (−0.25 V). The linear regression equation between oxidation peak current and scanning rate of the eluted modified electrode was *y* = 5.0407*x* + 17.626, in which *R* ^2^ was .9948; and the linear regression equation between reduction peak current and scanning rate was *y* = −5.013*x* − 17.481, *R* ^2^ = .9938. The scanning rate has a good linear relationship with the oxidation peak and reduction peak current of CV, and current change is stable. It shows that the electrochemical sensor has a stable diffusion performance during the detection process. With the increase of scanning rate, the electron conduction speed increases, the number of electrons involved in conduction increases, and the peak current increases accordingly.

### 3.5 Analytical characteristics for CEX determination

**Figure 6:**
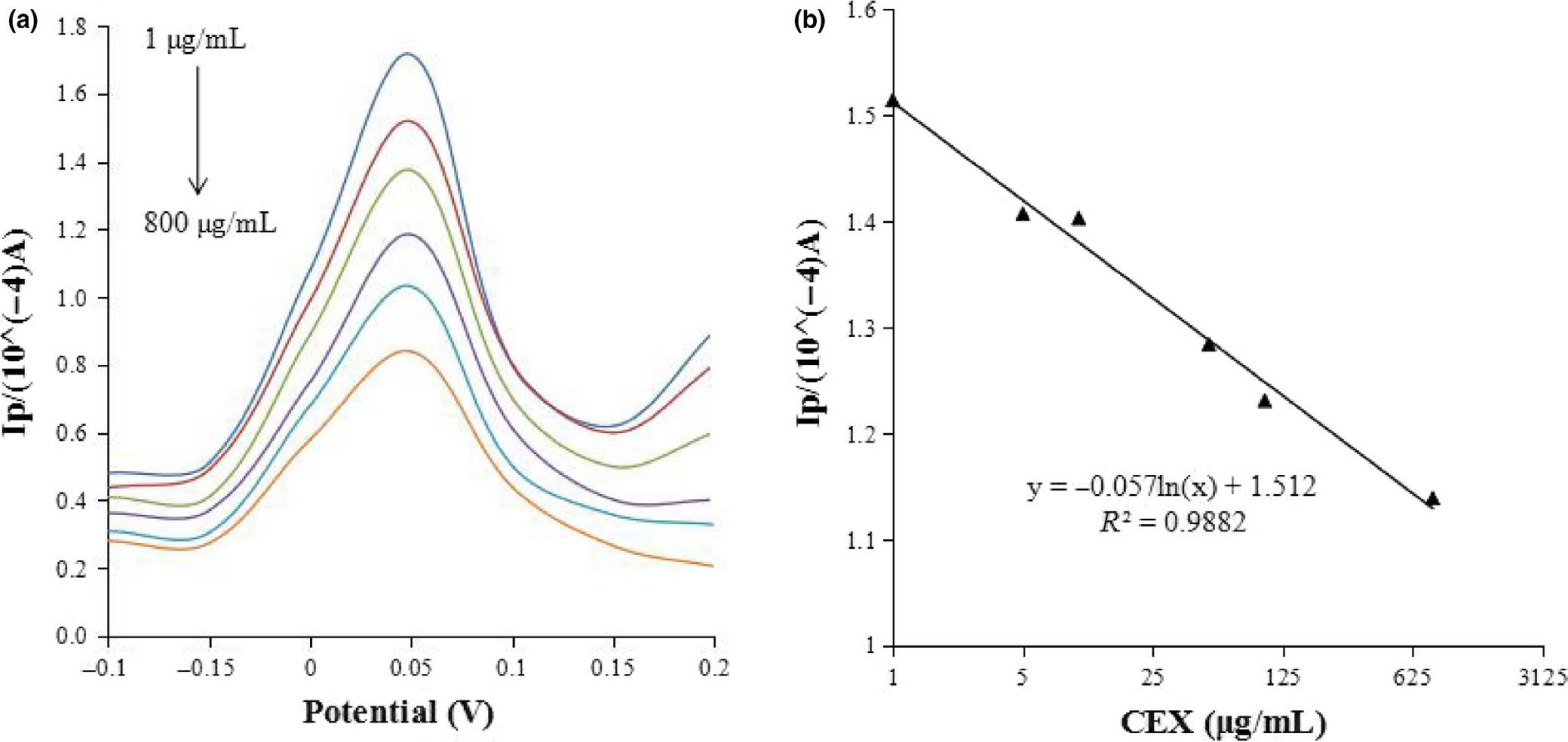
(a) DPV peak currents of 1 μg/ml, 5 μg/ml, 10 μg/ml, 50 μg/ml, 100 μg/ml, 600 μg/ml, and 800 μg/ml. (b) Plot of DPV peak currents versus different CEX concentrations. Potential range: 0.2–0.6 V; Pulse amplitude: 50 mV/s; Potential increment: 4 mV/s; Pulse width: 5 s; Pulse interval: 1 s

To evaluate the performance of the established sensor, the concentration of CEX was determined using the DPV with prepared CEX‐OVA/SWNTs‐COOH/CS/GCE. The calibration curve was linear in the range of 1 μg/ml to 800 μg/ml (Figure 6). The limit of detection was 45.7 ng/ml (*S/N* = 3). Compared with some of the detection methods based on CEX, as shown in Table 1, it is indicated that the proposed method was more sensitive than most of those previously studied.

**Table 1:** Results of DPV measurements of content in milk samples using the CEX‐OVA/SWNTs‐COOH/CS/GCE (*n* = 3)

	Added (μg/kg)	Milk		Yogurt		Milk powder	
		Found (μg/kg)	Recovery (%)	Found (μg/kg)	Recovery (%)	Found (μg/kg)	Recovery (%)
CEX	100	88.52	88.52	90.36	90.36	83.8	83.8
	1,000	908.2	90.82	825.8	82.58	867.5	86.75
	10,000	8,045	80.45	8,015	80.15	8,092	80.92

### 3.6 The specificity of the immunosensor

**Figure 7:**
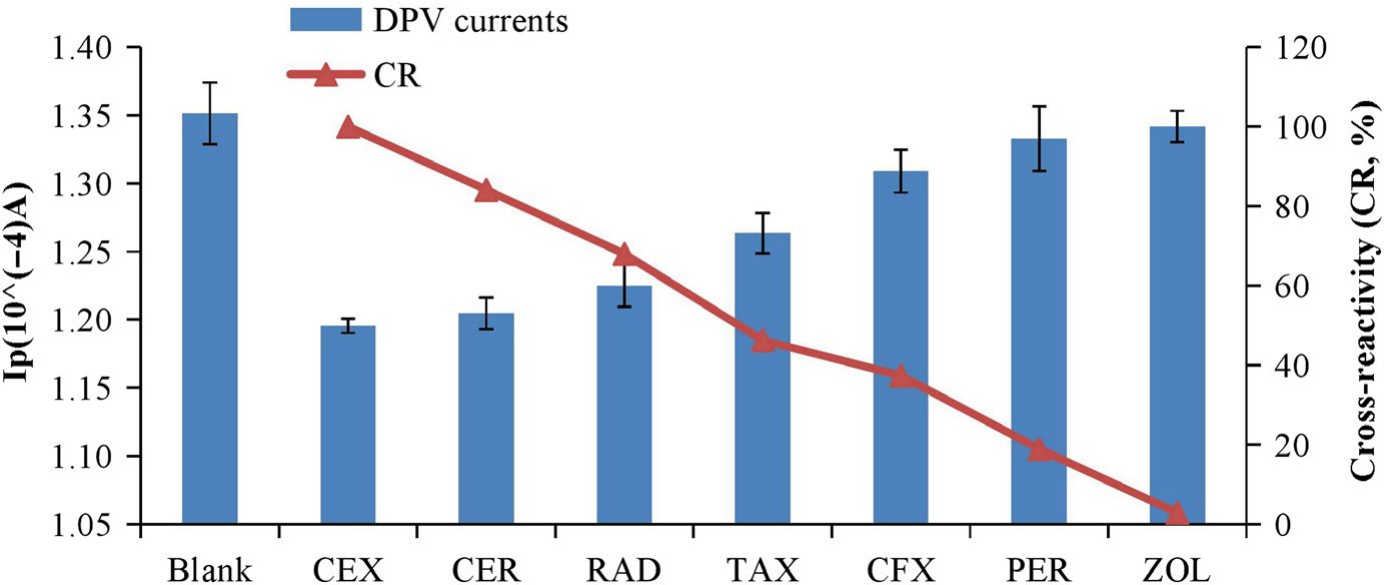
The current response of the immunosensor to CEX, CER, RAD, TAX, CFX, PER, and ZOL at 200 μl concentration, and the cross‐reactivity (CR) of CEX to the six other cephalosporins

In order to investigate the specificity of the immunosensor, CEX and six other cephalosporins of the same concentration including CER, RAD, CFX, PER, TAX, and ZOL were simultaneously assayed at the same concentration. The results of the DPV analysis are shown in Figure 7. Compared with the blank value, the ability to reduce peak current from high to low is as follows: cefalexin, cefadroxil, cefradine, cefotaxime, cefuroxime, cefoperazone, and cefazolin. To further assess the selectivity of the method, the cross‐reactivity (CR) of CEX to the six other cephalosporins were estimated. The results of CR between CEX and different cephalosporins were shown in Figure 7, and the CR values were calculated according to the equation.

**Figure 8:**
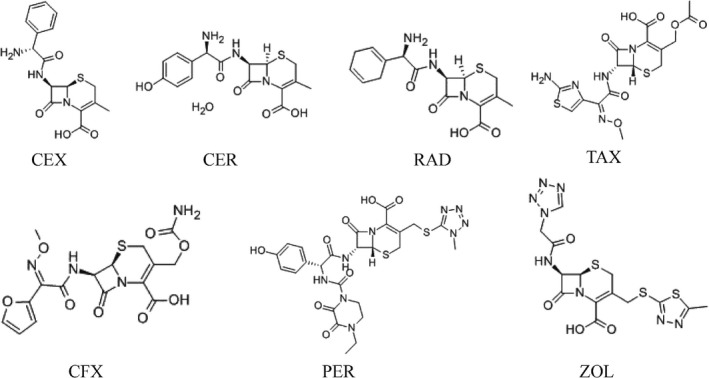
The molecular structure diagrams of CEX, CER, RAD, TAX, CFX, PER, and ZOL

The molecular structure of these seven cephalosporin antibiotics is shown in Figure 8. The substituents of these seven cephalosporins are different. Different atoms or groups in the organic molecule change the density of the bonding electron cloud in various ways, which does affect the organic reaction mechanism (Li et al., 2017).

The substituent of CEX is d‐phenyl glycine, the side chain group of CER is d‐phenyl glycine, and the substituent of RAD is 2‐amino‐2‐(1,4‐cyclohexadienyl) (Manjunatha, Shivappa Suresh, Savio Melo, D'Souza, & Venkatarangaiah Venkatesha, 2012). The substituent group is relatively simple. The structure of CER and RAD are less different from CEX. CER and RAD have a high cross‐reactivity rate.

The side chain of TAX is substituted by a sulfur‐containing group, and the side chain of aminothiazoline is closely related to its activity. The side chain of cis‐methoxyimino‐2‐aminothiazole is introduced into the side chain of TAX. The 2‐aminothiazole ring has a strong ability to penetrate the outer membrane and affinity with penicillin‐binding protein (PBPs), the methoxy group increases steric hindrance and its polar group thiol group makes it more stable to β‐lactamase (Gholivand, Torkashvand, & Malekzadeh, 2012). The substituent of CFX is connected to the methoxy group, and its stability is improved. The side chain of PER is replaced by the N‐methyltetrazole side chain (the MTT side chain), and the substituent of PER is complex. Its stability increases. The substituent of ZOL is N‐methyl thiadiazole side chain (MDT side chain) (Barbosa, Araujo, & Ferreira, 2012; Yang, Zhao, & Zeng, 2014), the steric hindrance increases. The structure of TAX, CFX, PER, and ZOL are quite different from CEX, so the cross‐reaction rate is low.

### 3.7 The stability of the immunosensor

**Figure 9:**
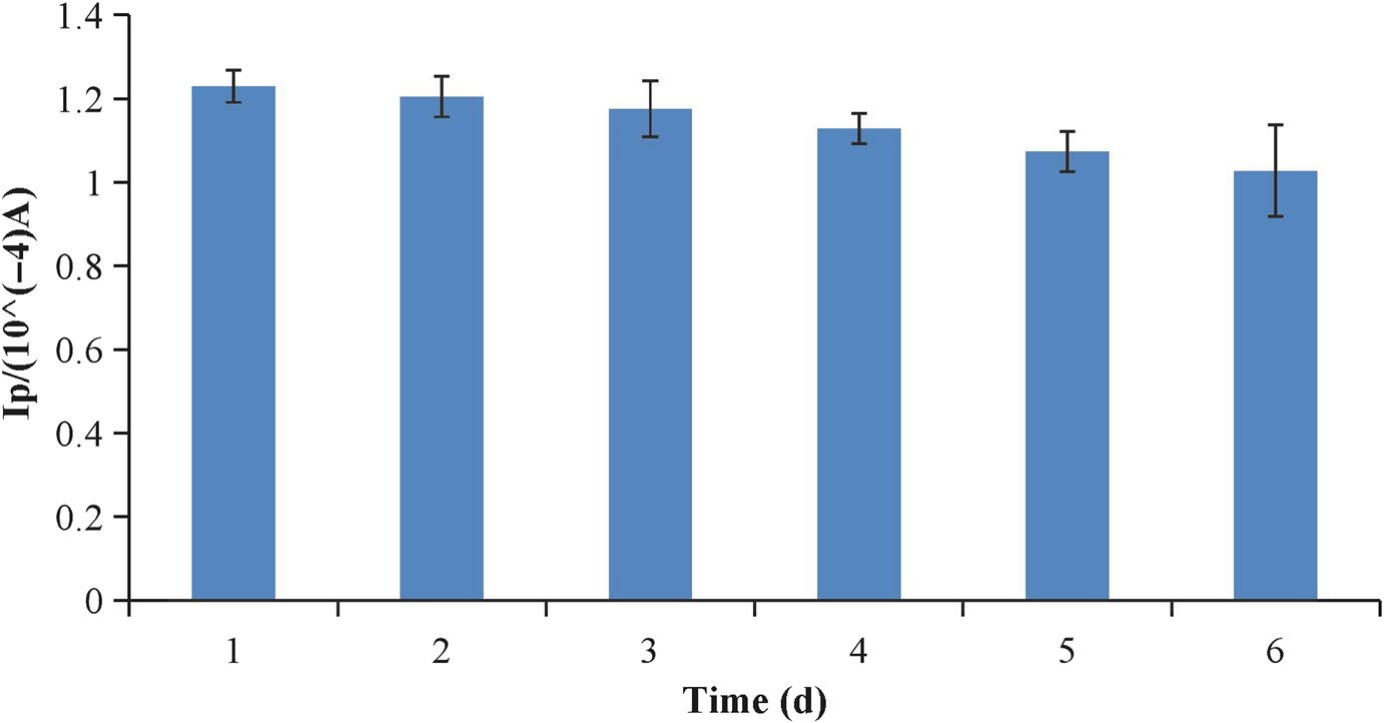
Current value changes in 6 days

To further ascertain the reproducibility of the experimental results, six different HRP‐anti‐antibody/anti‐CEX/CEX‐OVA/SWNTs‐COOH/CS/GCEs were tested toward the oxidation of 50 μmol/L CEX. The peak currents obtained by the six independent electrodes showed RSD of 8.6%, confirming that the sensor is reproducible. The stability of the HRP‐anti‐antibody/anti‐CEX/CEX‐OVA/SWNTs‐COOH/CS/GCE was also tested. After measurements, the modified electrode was stored at 4°C. As seen from Figure 9, the peak current intensity only decreased by 10.7% for the sensor after 6 days. The RSD (*n* = 5) for all these species was less than 11%. These results mentioned above indicated that the modified electrode possesses good reproducibility and good stability.

### 3.8 Application to actual sample determination

**Figure 10:**
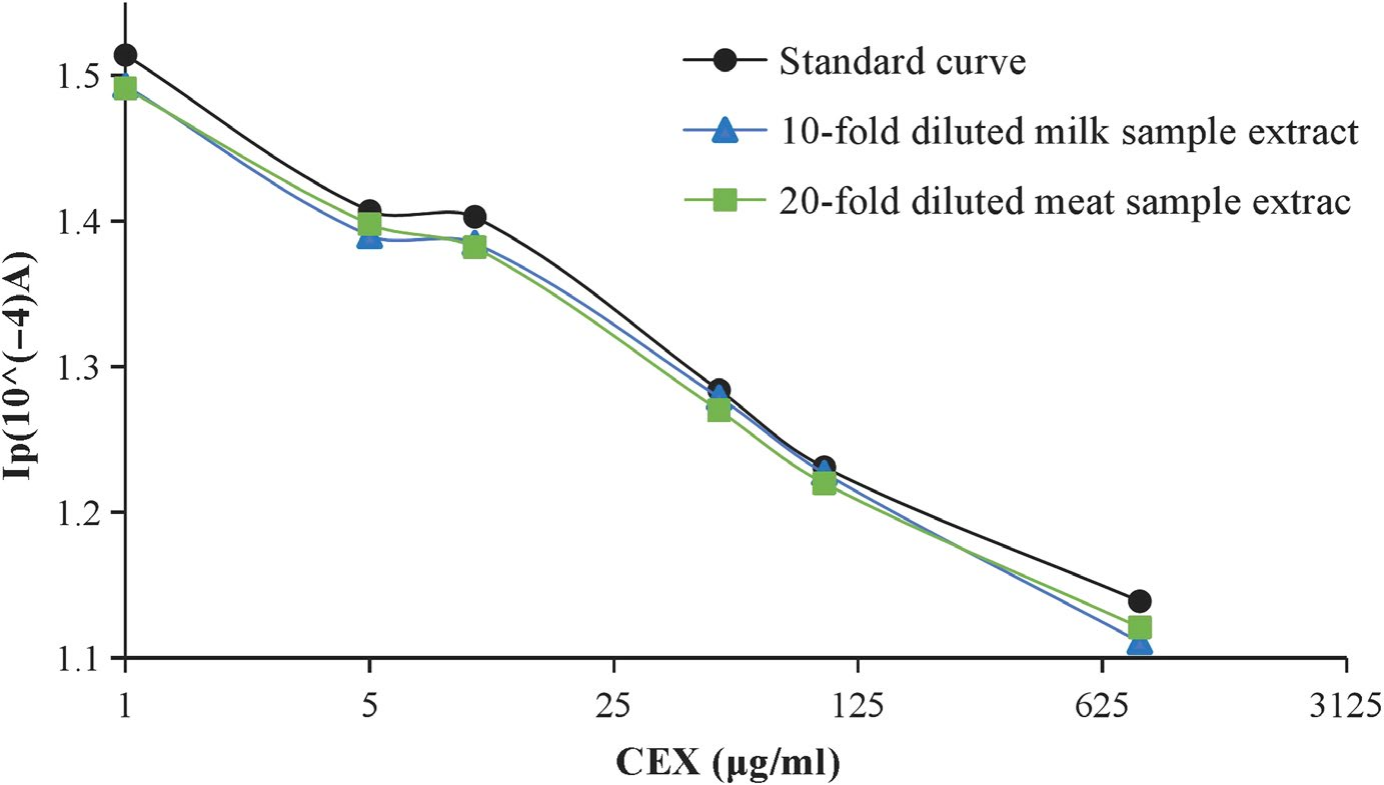
Comparison of the standard and matrix curves of meat and milk samples

Different tissue samples were treated according to the methods described in Section 2.4, as shown in Figure 10. There were some differences but only a little between the standard curve in working buffer and the adjusted standard curves which were built in two different sample extracts. It is indicated that the organic reagents in the diluted extract and the matrix effect exert little influence on the detection performance of the developed method. The adjusted standard curves were used for the real sample analysis in the following studies.

In order to validate the feasibility and sensibility of the developed immunosensor on detecting CEX, the content of CEX in milk samples, and meat samples were determined using the spiked recoveries method. The measurement results in Tables 1 and 2 indicated its good recoveries at three levels, ranging from 80.15% to 94.04%. Therefore, after improvement, the method could be regarded as a potential alternative for quantitative determination of CEX.

**Table 2:** Results of DPV measurements of content in meat samples using the CEX‐OVA/SWNTs‐COOH/CS/GCE (*n* = 3)

	Added (μg/kg)	Beef		Prawn		Fish	
		Found (μg/kg)	Recovery (%)	Found (μg/kg)	Recovery (%)	Found (μg/kg)	Recovery (%)
CEX	100	82.07	82.07	88.23	88.23	81.33	81.33
	1,000	804.1	80.41	940.37	94.04	845.5	84.55
	10,000	7,539.4	75.39	8,727	87.27	8,503	85.03

The comparisons between this electrochemical immunosensor and other recently reported methods for CEX were shown in Table 3. The LODs of these methods were ranging from 1.8 to 288 ng/ml, and our developed method exhibited a LOD of 45.7 ng/ml, which was lower than the MRLs of CEX in different food samples. So this method can meet the need for determination of CEX. In addition, the recoveries were better than most of other methods. This simple, reasonable, and economical method offers a new way for rapid and sensitive detection of CEX.

**Table 3:** Comparison of the developed method with several methods for CEX detection

Methods	LODs (ng/ml)	Recovery (%)	Comments	Ref.
Electrochemical sensors	35	92–96	High sensitivity, but need electrodes modification step	Feier, Gui, Cristea, and Săndulescu (2017)
Spectrofluorimetry	288	96–101	Low sensitivity compared to this work	Li et al. (2014)
Fluorescence polarization immunoassay	1.8	88–115	High sensitivity, and good reproducibility	Zhang, Wang, Mi, Wenren, and Wen (2014)
Spectrophotometric	168	96.04–102.2	Low sensitivity compared to this work	Ali Ahmed, Elbashir, and Aboul‐Enein (2015)
HPLC‐DAD	4	79–83	High sensitivity, but need multiple instrument operations	Camara et al. (2013)
This work	45.7	80.15–94.04	Convenient and accurate	–

## 4 CONCLUSIONS

In conclusion, this study described a sensitive, specific, and simple electrochemical immunosensor for detection of CEX by integrating SWNTs‐COOH/CS with enzymatic signal readout. The developed approach embodied its acceptable fabrication reproducibility and a high level of specificity and sensitivity for the detection of CEX at levels of 1–800 μg/ml. The sensor might further furnish a versatile and forceful tool to guarantee food safety.

## CONFLICT OF INTEREST

None.

## ETHICAL APPROVAL

This research did not include any human subjects and animal experiments.
